# The Climate of Algiers

**Published:** 1901-04-27

**Authors:** 


### THE CLIMATE OF ALGIERS.

On Tuesday Dr. Symes Thompson, Gresham Professor of Medicine, delivered to a large audience the first of four lectures on this subject at Gresham College. The lecturer described the physical basis of climate generally, and by means of a map of the world traced the chief isotherms and the causes which determined their course while passing across the world. For convalescents, he said, a climate should be chosen which showed no great variations of heat and cold. Humidity contributed to equability, whereas dryness was usually accompanied by marked changes in the day and night temperatures. But it was important to remember that extremes of temperature contributed greatly to the sturdiness of a race. Countries on the Mediterranean seaboard had a typically temperate climate. On the North, including the Riviera, shelter from the cold winds was afforded by the Maritime Alps. The southern shores had the advantage of the humidity of the Mediterranean and the dryness of the Sahara. The salt in the air at the seaboard not only tempered the humidity but had in itself a distinct medicinal value. The lecturer compared the saltness of the Baltic at different parts with that of the Dead Sea, and explained the cause of differences. Salt was also present in the sand of the desert, as could be seen by minute incrustations. A wonderful provision of nature was shown in the case of a plant which grew in parts where there was neither dew nor rainfall. The plant had long roots, which sucked up a small amount of moisture, and with it some of the saline constituents of the soil, which were deposited on the leaf. This salt absorbed watery vapour from the air, and so kept the plant moist.

Drainage, aspect, and elevation were next touched upon. Flat hollow situations were shown to be conducive to stagnation and humidity, and therefore unhealthiness; whereas, with free drainage the reverse was the case. The peculiar benefits to be derived from the Algerian climate were fully explained, and an admirable series of photographs was shown illustrating the scenery and native customs. The lecturer then proceeded to compare continental and insular climates, and showed that in the former case there were long periods of cold followed by similarly consistent heat, whereas insular climates were characterised by great instability of weather. The valley of the Nile was a typical example of climates characterised by floods at one time and great dryness at others. It was the alluvium of the valley of the Nile which had made Egypt what it had been throughout history. This alluvium consisted of very fine dust, which, as Professor Drummond had shown, was due to the ant breaking up the soil into very minute particles, and that gave it its great value. Darwin had similarly made it clear that the earthworm was essential to the aëration and fertilisation of our soil. Unless there was a high wind, the sand of the Sahara was not a source of much inconvenience, as its particles were large compared with those of alluvium. If one kicked the sand of the Sahara the resulting dust soon settled; but dry alluvium so treated caused a cloud which remained suspended for some time. Very few noxious germs were found in the sand. Many of the natives lived in roofless houses, but in the old town of Algiers, where there was a considerable rainfall, some roofs were to be seen, and in these houses overcrowding occurred, a family sometimes being found in a room from 4 feet to 6 feet square. It was not, therefore, surprising that few of the children lived beyond 5 years. The equalising effect of proximity to the sea was next dealt with, and the well-known explanation that water retains its heat much longer than land was lucidly given. At Algiers the climate tended towards a lazy life, so that considerable responsibility rested on those who sent reposeful people to such a place! On the other had, Switzerland caused a desire for activity, and in that country one felt it was the right thing to do much mountain climbing. The habits of the natives of a particular place accurately indicated the climatic tendency; a warm humid climate with abundant vegetation rendering arduous work unnecessary for a living; whereas in colder and more arid regions much energy was required for subsistence. A striking example of the former was seen in the case of Egypt. It was a rule there that a British officer was not to strike a native; but it was found that the native would only work when chastised, and therefore it became necessary to sub-let Government work.

In conclusion the lecturer spoke of mineral ingredients in soils, and alluded to an arsenical magnesian limestone the waters from which were valuable for certain skin diseases.

